# ﻿A new genus and nine species of jumping spiders from Hainan Island, China (Araneae, Salticidae)

**DOI:** 10.3897/zookeys.1118.89337

**Published:** 2022-08-18

**Authors:** Cheng Wang, Shuqiang Li

**Affiliations:** 1 Guizhou Provincial Key Laboratory for Biodiversity Conservation and Utilization in the Fanjing Mountain Region, Tongren University, Tongren, Guizhou 554300, China Tongren University Tongren China; 2 Ministry of Education Key Laboratory for Ecology of Tropical Islands, College of Life Sciences, Hainan Normal University, Haikou 571158, China Hainan Normal University Haikou China; 3 Institute of Zoology, Chinese Academy of Sciences, Beijing 100101, China Institute of Zoology, Chinese Academy of sciences Beijing China

**Keywords:** Morphology, new combination, new taxa, rainforest, salticid, taxonomy

## Abstract

A new genus and eight new species of jumping spiders from Hainan Island, China are reported. *Pengmarengo***gen. nov.** is erected to accommodate the type species *P.yangi***sp. nov.** (♂♀). Further *Pengmarengo***gen. nov.** species including *P.chelifer* (Simon, 1990), **comb. nov.** (transferred from *Philates* Simon, 1900), *P.elongata* (Peng & Li, 2002), **comb. nov.** (transferred from *Tauala* Wanless, 1988), and two species transferred from *Indomarengo* Benjamin, 2004: *P.yui* (Wang & Li, 2020), **comb. nov.**, and *P.wengnan* (Wang & Li, 2022), **comb. nov.** Another seven new jumping spider species are described from Hainan: *Iruraliae***sp. nov.** (♂), *I.mii***sp. nov.** (♂♀), *Marengoganae***sp. nov.** (♂♀), *M.zhengi***sp. nov.** (♂♀), *Nungiatangi***sp. nov.** (♂♀), *Philateszhoui***sp. nov.** (♂♀), and *Toxeushainan***sp. nov.** (♂♀). The unknown female of the endemic species, *Irurapengi* Guo, Zhang & Zhu, 2011 is also described for the first time.

## ﻿Introduction

Hainan, the second-largest island in China, possesses a large number of tropical rainforests. The island has high species diversity as well as high ratio of endemism (Li 2020; Wang et al 2020; Li et al 2021; Yao et al 2021; Hong et al 2022; Zhu et al 2022). A series of research on jumping spiders from this island has reported 37 endemic species and increased the species number to 118 (Peng and Kim 1997; Peng and Li 2006; Guo et al. 2011; Zhou and Li 2013). However, nearly half (17) of endemic species are known only from a single-sex, and part of them are lacking distinct diagnostic drawings indicating the jumping spider of this island remains poorly studied (WSC 2022). In our recent study of salticid samples from Hainan Island, eight species belonging to six genera (including a new genus) are recognized as new to science, and the unknown female of *Irurapengi* Guo, Zhang & Zhu, 2011 has been found.

## ﻿Materials and methods

Specimens were collected by beating shrubs or hand collecting in the tropical rainforest of Hainan Island, China. They were preserved in 75% ethanol for morphological study and in absolute ethanol for molecular study. Specimens are deposited in the Institute of Zoology, Chinese Academy of Sciences in Beijing (**IZCAS**), China, and Tongren University (**TRU**) in Tongren, China. Methods follow those of Wang and Li (2021).

All measurements are given in millimeters. Leg measurements are given as: total length (femur, patella, tibia, metatarsus, tarsus). References to figures in the cited papers are listed in lowercase type (fig. or figs), and figures in this paper are noted with an initial capital (Fig. or Figs). Abbreviations used in the text and figures are as follows:

**AERW** anterior eye row width;

**AME** anterior median eye;

**ALE** anterior lateral eye;

**AG** accessory gland;

**AR** atrial ridge;

**AS** anterior chamber of spermatheca;

**At** atrium;

**BP** basal epigynal plate;

**CA** cymbial apophysis;

**CD** copulatory duct;

**CO** copulatory opening;

**DCP** dorsal cymbial process;

**DTA** dorsal tibial apophysis;

**E** embolus;

**EC** embolic coil;

**EFL** eye field length;

**FD** fertilization duct;

**JS** junction duct of spermathecae;

**H** epigynal hood;

**MS** median septum;

**MiS** median chamber of spermatheca;

**PERW** posterior eye row width;

**PED** process of embolic disc;

**PLE** posterior lateral eye;

**PS** posterior chamber of spermatheca;

**PTA** prolateral tibial apophysis;

**RTA** retrolateral tibial apophysis;

**S** spermatheca;

**SD** sperm duct;

**St** stiffener;

**TF** tibial flange.

## ﻿Taxonomy

### ﻿Family Salticidae Blackwall, 1841

#### ﻿Genus Irura

Taxon classificationAnimaliaAraneaeSalticidae

Peckham & Peckham, 1901

1767288E-AC46-5AB7-BC05-94BA6A08C00A

##### Type species.

*Irurapulchra* Peckham & Peckham, 1901 from Sri Lanka by original designation.

##### Comments.

The genus *Irura* Peckham & Peckham, 1901 is placed in the subtribe Simaethina Simon, 1903 together with other 12 genera and is represented by 18 species mainly distributed from East and Southeast Asia (Maddison 2015; WSC 2022). It is rather poorly understood because the generotype is known from single-sex and lacks key diagnostic drawings. According to the morphological character, the genus is similar to *Stertinius* Simon, 1890 in having the PME closer to AME than to PLE, three pairs of conspicuous muscle depressions on the dorsum of abdomen (Logunov 2022), but it differs by the sub-oval carapace and the well-developed (extending exceed the cymbial base) cymbial apophysis mostly possesses a pointed terminus, whereas almost square carapace, less-developed (not extending exceed the cymbial base) cymbial apophysis without pointed terminus in *Stertinius* (see Metzner 2022).

#### ﻿ Irura liae sp. nov.

Taxon classificationAnimaliaAraneaeSalticidae

B4489F34-181F-5A77-86F5-670CD529647F

https://zoobank.org/CCC36061-F7D4-4FB4-BDA0-1D67CF4DA655

Fig. 1

##### Type material.

***Holotype*** ♂ (TRU-JS 0622), China: Hainan: Lingshui County, Diaoluoshan National Nature Reserve, 01–05.v.2021, F.E. Li leg.

##### Etymology.

The specific name is a patronym of Ms Feng’E Li, the collector of the type specimen; noun (name) in genitive case.

##### Diagnosis.

**Figure 1.**
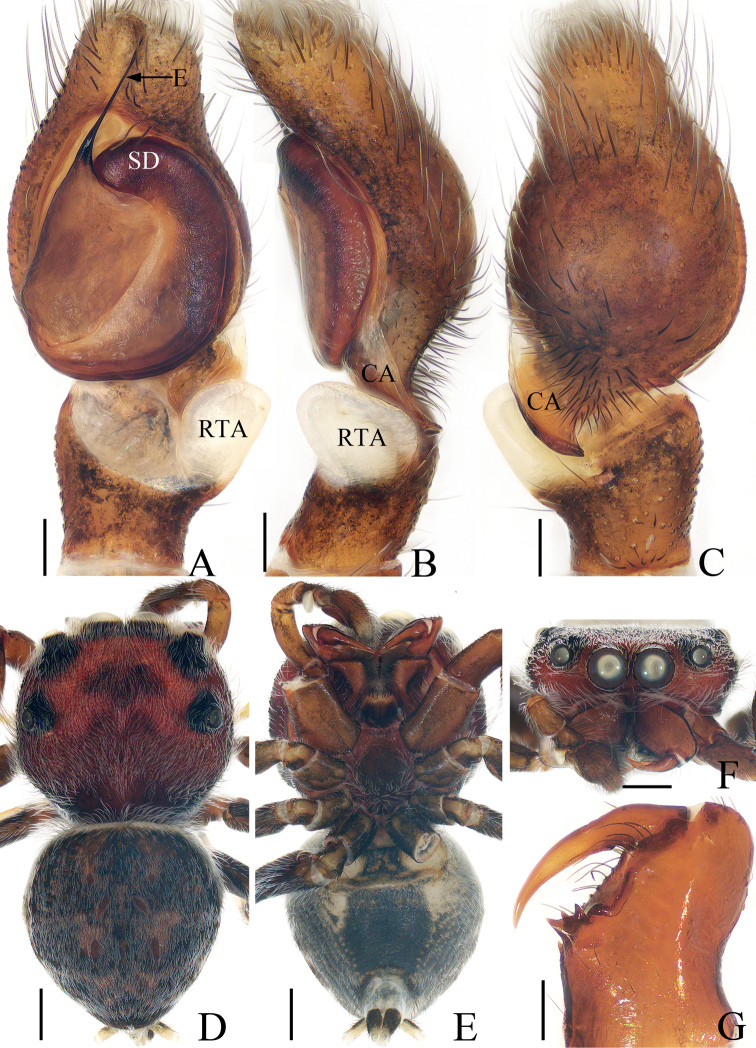
*Iruraliae* sp. nov., male holotype **A** palp, ventral **B** ditto, retrolateral **C** ditto, dorsal **D** habitus, dorsal **E** ditto, ventral **F** carapace, frontal **G** chelicera, posterior. Scale bars: 0.1 mm (**A–C, G**); 0.5 mm (**D–F**). Abbreviations: CA – cymbial apophysis; E – embolus; RTA – retrolateral tibial apophysis; SD – sperm duct.

*Iruraliae* sp. nov. closely resembles *I.bidenticulata* Guo, Zhang & Zhu, 2011 known from Hainan, and Hongkong of China in having a short embolus and a weakly sclerotized RTA, but it can be easily distinguished by the following characters: (1) the RTA is almost disciform in ventral view (Fig. 1A), whereas it is elongated in *I.bidenticulata* (Guo et al. 2011: fig. 8); (2) the embolus is ~ 3/5 of the bulb length (Fig. 1A), whereas it is ca. as long as the bulb in *I.bidenticulata* (Guo et al. 2011: fig. 8).

##### Description.

**Male** (Fig. 1). Total length 3.89. Carapace 2.05 long, 2.17 wide. Abdomen 2.06 long, 1.96 wide. Eye sizes and inter-distances: AME 0.44, ALE 0.22, PLE 0.21, AERW 1.67, PERW 2.03, EFL 0.92. Leg measurements: I 7.56 (2.38, 1.60, 1.70, 1.13, 0.75), II 3.51 (1.25, 0.48, 0.75, 0.63, 0.40), III 3.02 (1.01, 0.48, 0.50, 0.63, 0.40), IV 3.71 (1.25, 0.63, 0.68, 0.75, 0.40). Carapace almost oval, red-brown to dark brown, covered with dense, pale setae, with dark double-humped patch medially in eye field. Chelicerae red-brown, with two promarginal teeth and one retromarginal fissidental tooth with two cusps. Endites longer than wide, with dense, dark setae on inner margins. Labium almost linguiform, paler distally, bearing dark setae at anterior edge. Sternum red-brown to dark brown, bearing pale setae of varying lengths. Legs I robust, with two pairs of macrosetae ventrally on tibiae and metatarsi, respectively; other legs pale to brown. Abdomen oval, dorsum dark brown, covered with pale, thin setae, with three pairs of muscle depressions medially, and transverse, undulate, earthy yellow streaks posteriorly, covered entirely by a large scutum; venter dark brown medially, with anterolateral pale areas. Palp (Fig. 1A–C): tibia longer than wide, with weakly sclerotized, disciform RTA; cymbium acutely narrowed distally, with baso-retrolateral apophysis slightly curved medially and slightly pointed at distal end; bulb flat, almost round, with tapered sperm duct extending along margin; embolus filiform, strongly sclerotized, straight, originates at ~ 10:30 o’clock position on bulb, ~ 3/5 the bulb length.

**Female**. Unknown.

##### Distribution.

Only known from the type locality on Hainan Island, China.

#### ﻿ Irura mii sp. nov.

Taxon classificationAnimaliaAraneaeSalticidae

EDBA61A3-21C2-57CF-B10E-0FD71AE9D1D9

https://zoobank.org/F52D4E03-401D-4E2B-8BA5-45FD166FB7BD

Figs 2 , 3

##### Type material.

***Holotype*** ♂ (IZCAS-Ar43164), China: Hainan: Wuzhishan City, Wuzhi Mountain National Nature Reserve, hillside (18°53.83'N, 109°41.88'E, ca. 1590 m), 08.iv.2009, G. Tang leg. ***Paratypes*** 3♂9♀ (IZCAS-Ar43165–43176), same data as holotype; 2♂ (IZCAS-Ar43177–43178), hillside (18°53.84'N, 109°41.51'E, ca. 1210 m), same date and collector as holotype; 4♀ (IZCAS-Ar43179–43182), hillside (18°53.85'N, 109°41.89'E, ca. 1430 m), 09.iv.2009, G. Tang leg.; 1♂1♀ (TRU-JS 0623–0624), Ledong County, Jianfengling National Nature Reserve, Peak Mountain (18°43.11'N, 108°52.32'E, ca. 1400 m), 16.iv.2019, C. Wang & Y.F. Yang leg.

##### Etymology.

The specific name is a patronym of Dr. Xiaoqi Mi, who greatly helped us with this research; noun (name) in genitive case.

##### Diagnosis.

The male of *Iruramii* sp. nov. can be easily distinguished from other congeners by the presence of PTA, and the distally semi-circled, filiform embolus, whereas absent, and not circled, flagelliform in others (see Metzner 2022). The female of this new species resembles *I.hamatapophysis* (Peng & Yin, 1991) known from Hunan of China in having the copulatory openings located at the posterior margin and the elongated junction ducts of spermathecae, but it can be easily distinguished by the copulatory ducts, which are longer and connected to the middle of the junction ducts of the spermathecae, and by the oval posterior chamber of the spermathecae (Fig. 3B), whereas the copulatory ducts are shorter, connected to the distal portions of the junction ducts of the spermathecae, and the posterior chamber of the spermathecae are eggplant-shaped in *I.hamatapophysis* (Peng and Yin 1991: fig. 1H, I).

##### Description.

**Figure 2.**
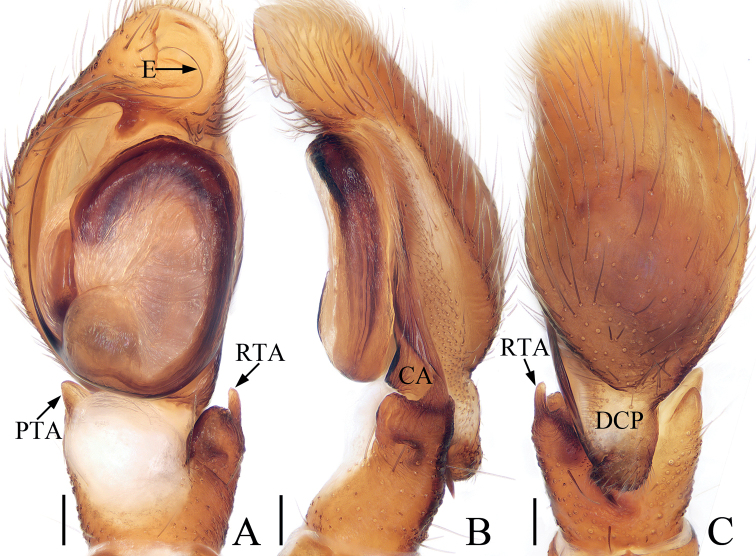
Male palp of *Iruramii* sp. nov., holotype **A** ventral **B** retrolateral **C** dorsal. Scale bars: 0.1 mm. Abbreviations: CA – cymbial apophysis; DCP – dorsal cymbial process; E – embolus; PTA – prolateral tibial apophysis; RTA – retrolateral tibial apophysis.

**Figure 3.**
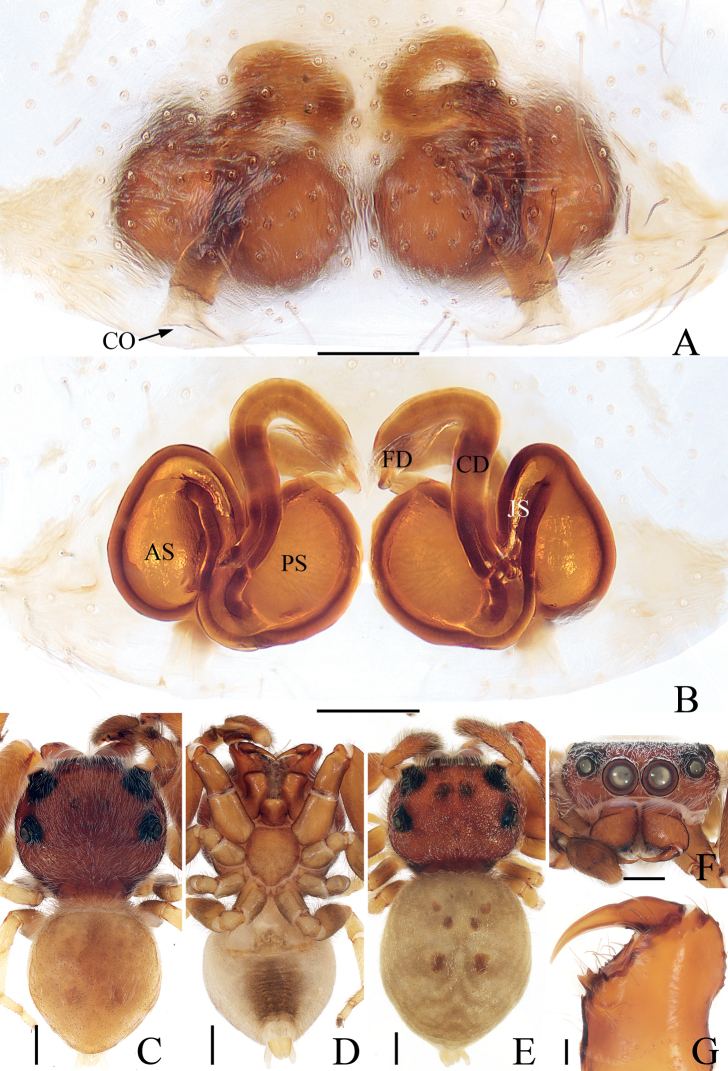
*Iruramii* sp. nov., male holotype and female paratype **A** epigyne, ventral **B** vulva, dorsal **C** holotype habitus, dorsal **D** ditto, ventral **E** female paratype habitus, dorsal **F** holotype carapace, frontal **G** holotype chelicera, posterior. Scale bars: 0.1 mm (**A, B, G**); 0.5 mm (**C–F**). Abbreviations: AS – anterior chamber of spermatheca; CD – copulatory duct; CO – copulatory opening; FD – fertilization duct; JS – junction duct of spermathecae; PS – posterior chamber of spermatheca.

**Male** (Figs 2, 3C, D, F, G). Total length 3.67. Carapace 1.83 long, 1.98 wide. Abdomen 1.89 long, 1.75 wide. Eye sizes and inter-distances: AME 0.45, ALE 0.26, PLE 0.21, AERW 1.61, PERW 1.86, EFL 0.92. Leg measurements: I 6.02 (1.83, 1.30, 1.38, 0.88, 0.63), II 3.22 (1.08, 0.58, 0.65, 0.58, 0.33), III 2.79 (0.93, 0.45, 0.53, 0.55, 0.33), IV 3.31 (1.10, 0.55, 0.70, 0.63, 0.33). Carapace almost oval, red-brown, setose, with pair of round, dark spots medially in eye field. Fovea indistinct. Chelicerae red-yellow, with two promarginal teeth and one retromarginal fissidental tooth. Endites longer than wide, bearing dense setae on distal portions of inner margins. Labium colored as endites. Sternum almost oval, with straight anterior margin. Legs I robust, with two pairs of macrosetae ventrally on tibiae and metatarsi, respectively; other legs pale to brown. Abdomen oval, dorsum covered entirely by large scutum, with white setae and three pairs of muscle depressions; venter dark brown medially, with brown, thin setae. Palp (Fig. 2A–C): tibia slightly wider than long in ventral view, with sub-triangular prolateral apophysis and stout, broad RTA acutely narrowed to triangle shape distally in retrolateral view; cymbium longer than wide, with broad, irregular baso-dorsal process and spine-shaped retrolateral apophysis extending exceed dorsal process distally; bulb oval, flat, with sperm duct extending along margin; embolus slender, filiform, originating at ~ 7: 30 o’clock position of bulb, coiled into a semi-circle distally.

**Female** (Fig. 3A, B, E). Total length 3.68. Carapace 1.49 long, 1.76 wide. Abdomen 2.30 long, 2.03 wide. Eye sizes and inter-distances: AME 0.41, ALE 0.20, PLE 0.17, AERW 1.39, PERW 1.69, EFL 0.81. Leg measurements: I 4.70 (1.63, 1.03, 1.03, 0.68, 0.33), II 3.10 (1.03, 0.58, 0.63, 0.53, 0.33), III 2.70 (0.90, 0.45, 0.50, 0.55, 0.30), IV 3.32 (1.13, 0.53, 0.68, 0.65, 0.33). Habitus (Fig. 3E) similar to that of male except with several herringbone-shaped stripes posteriorly and lacks a scutum on the dorsum of abdomen. Epigyne (Fig. 3A, B): wider than long; copulatory openings located at posterior margin, almost round, separated from each other by > 2 × the width of posterior chamber of spermathecae; copulatory ducts long, twisted, connected to the middle of junction ducts of spermathecae; spermathecae divided into two oval chambers; fertilization ducts lamellar, originate from anterior parts of posterior chamber of spermathecae.

##### Distribution.

Only known from the type locality on Hainan Island, China.

#### ﻿ Irura pengi

Taxon classificationAnimaliaAraneaeSalticidae

Guo, Zhang & Zhu, 2011

5492A84F-1E29-5747-AF1D-1C76C267899D

Figs 4 , 5

Irura pengi Guo, Zhang & Zhu, 2011: 91, figs 11–16 (♂, holotype, not examined).

##### Material examined.

2♂3♀ (TRU-JS 0625–0629), China: Hainan: Ledong County, Jianfengling National Nature Reserve, Tianchi (18°44.45'N, 108°57.49'E, ca. 860 m), 11.iv.2019, C. Wang & Y.F. Yang leg.

##### Diagnosis.

**Figure 4.**
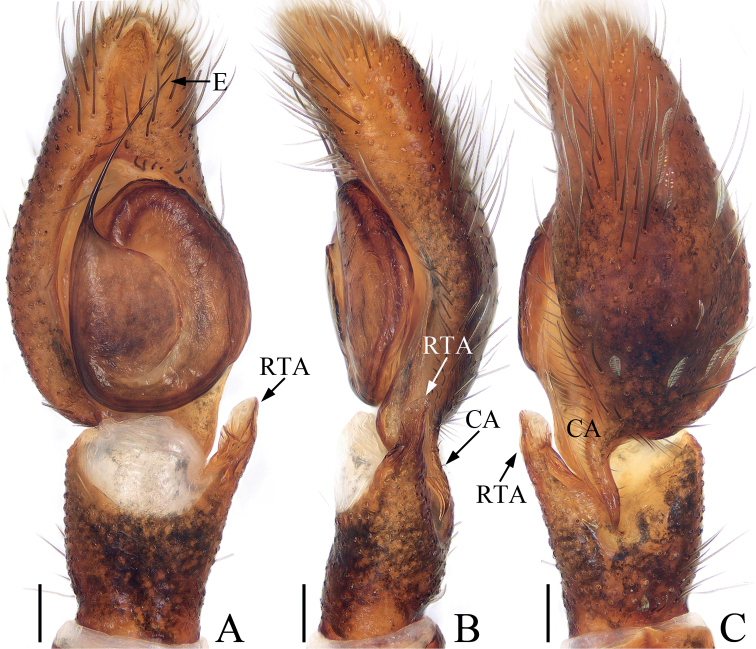
Male palp of *Irurapengi* Guo, Zhang & Zhu, 2011 **A** ventral **B** retrolateral **C** dorsal. Scale bars: 0.1 mm. Abbreviations: CA – cymbial apophysis; E – embolus; RTA – retrolateral tibial apophysis.

**Figure 5.**
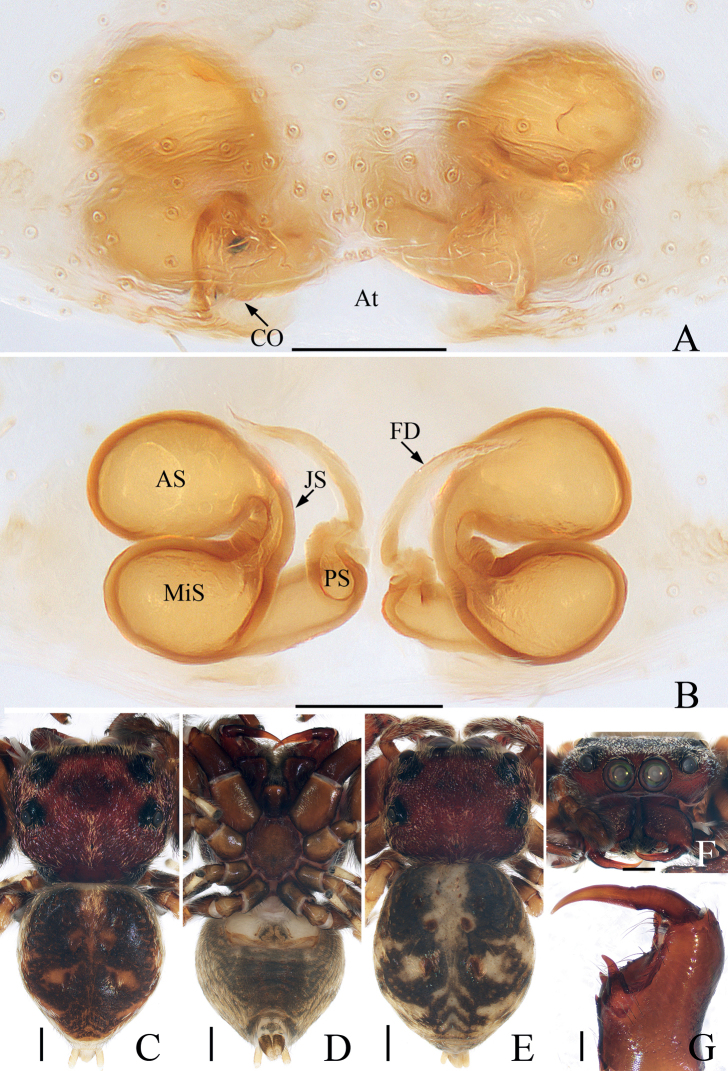
*Irurapengi* Guo, Zhang & Zhu, 2011 **A** epigyne, ventral **B** vulva, dorsal **C** male habitus, dorsal **D** ditto, ventral **E** female habitus, dorsal **F** male carapace, frontal **G** male chelicera, posterior. Scale bars: 0.1 mm (**A, B, G**); 0.5 mm (**C–F**). Abbreviations: AS – anterior chamber of spermatheca; At – atrium; CO – copulatory opening; FD – fertilization duct; MiS – median chamber of spermatheca; JS – junction duct of spermathecae; PS – posterior chamber of spermatheca.

The male of *Irurapengi* Guo, Zhang & Zhu, 2011 resembles *I.trigonapophysis* (Peng & Yin, 1991) known from Fujian, and Guangdong of China in having a flagelliform embolus originating at ~ 10 o’clock position on the bulb, but it can be distinguished by the straight RTA and terminally curved retrolateral cymbial apophysis in retrolateral view (Fig. 4B), whereas the RTA is slightly curved dorsally and the retrolateral cymbial apophysis is straight in *I.trigonapophysis* (Peng 2020: fig. 127b–d). The species also resembles that of *I.uniprocessa* Mi & Wang, 2016 known from Yunnan of China by the similar copulatory organs, but it can be distinguished by the following characters: (1) the presence of RTA (Fig. 4A–C), which is absent in *I.uniprocessa* (Mi and Wang 2016: figs 1C–E, 2a, b); (2) the absence of epigynal hoods and the distance between the anterior chambers of spermathecae is more than their width (Fig. 5B), whereas present and the distance between the anterior chambers of spermathecae is < 1/2 their width in *I.uniprocessa* (Mi and Wang 2016: figs 1F, G, 2d, e).

##### Description.

**Male** (Figs 4, 5C, D, F, G). Total length 4.52. Carapace 2.06 long, 2.35 wide. Abdomen 2.50 long, 2.26 wide. Eye sizes and inter-distances: AME 0.50, ALE 0.29, PLE 0.24, AERW 1.91, PERW 2.21, EFL 1.12. Leg measurements: I 7.02 (2.13, 1.63, 1.58, 1.03, 0.65), II 3.98 (1.28, 0.75, 0.85, 0.70, 0.40), III 3.32 (1.08, 0.58, 0.63, 0.63, 0.40), IV 3.87 (1.33, 0.63, 0.81, 0.70, 0.40). Carapace sub-square, red-brown, covered with dense, iridescent scales, with an irregular dark patch medially in eye field. Chelicerae red-brown, with two promarginal teeth and one large retromarginal tooth. Endites longer than wide, with dense, dark brown setae at inner-distal margins. Labium colored as endites, bearing dense, dark setae at anterior edges. Sternum sub-oval, covered with thin setae. Legs I robust, with a single and two pairs of macrosetae ventrally on tibiae and metatarsi, respectively; other legs yellow to dark. Abdomen oval, dorsum orange-red to dark brown, with longitudinal orange band anteromedially, three pairs of muscle depressions and several pairs of irregular orange patches medially and laterally, and several herringbone-shaped streaks posteriorly, covered entirely by a big scutum; venter brown to dark brown. Palp (Fig. 4A–C): tibia longer than wide, with straight RTA ~ 1/2 the tibia length, and blunt apically in retrolateral view; cymbium elongated, setose, with tapered, baso-retrolateral apophysis curved distally to a pointed tip, reaches anterior 1/3 of tibia in retrolateral view; bulb flat, almost round; embolus flagelliform, tapered, originates at ~ 10 o’clock position on bulb, slightly shorter than bulb length.

**Female** (Fig. 5A, B, E). Total length 4.26. Carapace 1.58 long, 2.06 wide. Abdomen 2.74 long, 2.29 wide. Eye sizes and inter-distances: AME 0.48, ALE 0.26, PLE 0.21, AERW 1.74, PERW 2.03, EFL 0.97. Leg measurements: I 4.33 (1.40, 1.00, 0.85, 0.60, 0.48), II 3.11 (1.00, 0.53, 0.70, 0.50, 0.38), III 2.70 (0.88, 0.48, 0.48, 0.48, 0.38), IV 3.27 (1.13, 0.55, 0.63, 0.58, 0.38). Habitus (Fig. 5E) similar to that of male except pale and with less-developed retromarginal cheliceral tooth. Epigyne (Fig. 5A, B): wider than long, with broad, posteriorly-located atrium; copulatory openings beneath the antero-bilateral edge of atrium; copulatory ducts short, tapered, connected to the junction ducts of anterior and middle chambers of spermathecae; spermathecae divided into three oval chambers; fertilization ducts elongated, originate from anterior edges of the smallest posterior chamber of spermathecae.

##### Distribution.

Only known from Hainan Island, China.

#### ﻿Genus Marengo

Taxon classificationAnimaliaAraneaeSalticidae

Peckham & Peckham, 1892

7EBBAD96-5FC1-50A3-AB4E-688447C5B662

##### Type species.

*Marengocrassipes* Peckham & Peckham, 1892 from Sri Lanka by original designation.

##### Comments.

*Marengo*, a tribe Baviini genus, contains ten species distributed in India, Sri Lanka, Thailand, and China (Maddison 2015; WSC 2022). A recent re-defined of the genus was provided by Benjamin (2004), who diagnosed the genus by the presence of ventral, leaf-like scales on tibiae I and the accessory gland of copulatory ducts. However, even within a genus, the copulatory organs in the Ballini look alike and are very often useless for supraspecific diagnoses (Azarkina and Haddad 2020). And so, the above definition could not be accurate. Herein, the definition of the genus is not discussed, and we assigned the following two new species to *Marengo* because they are closely similar to some species of the genus.

#### ﻿ Marengo ganae sp. nov.

Taxon classificationAnimaliaAraneaeSalticidae

32FB8227-C048-52FD-9D65-9AAE9662EAD7

https://zoobank.org/3F140AF9-B89C-476A-8CF0-B8E0B0BD5053

Figs 6 , 7

##### Type material.

***Holotype*** ♂ (TRU-JS 0630), China: Hainan: Ledong County, Jianfeng Village, Jianfengling National Nature Reserve, Tianchi (18°44.90'N, 108°52.01'E, ca. 790 m), 12.viii.2020, X.Q. Mi et al. leg. ***Paratypes*** 1♂2♀ (TRU-JS 0631–0633), same data as holotype; 1♀ (IZCAS-Ar43183), Lingshui County, Diaoluoshan National Nature Reserve (18°43.44'N, 109°52.60'E, ca. 490 m), 10.viii.2010, G. Zheng leg.; 1♂1♀ (IZCAS-Ar43184–43185), Diaoluoshan National Nature Reserve, Luchang (18°43.39'N, 109°51.07'E, ca. 940 m), 10.viii.2010, G. Tang leg.

##### Etymology.

The specific name is a patronym of Mrs. Jiahui Gan, one of the collectors of the type specimens; noun (name) in genitive case.

##### Diagnosis.

**Figure 6.**
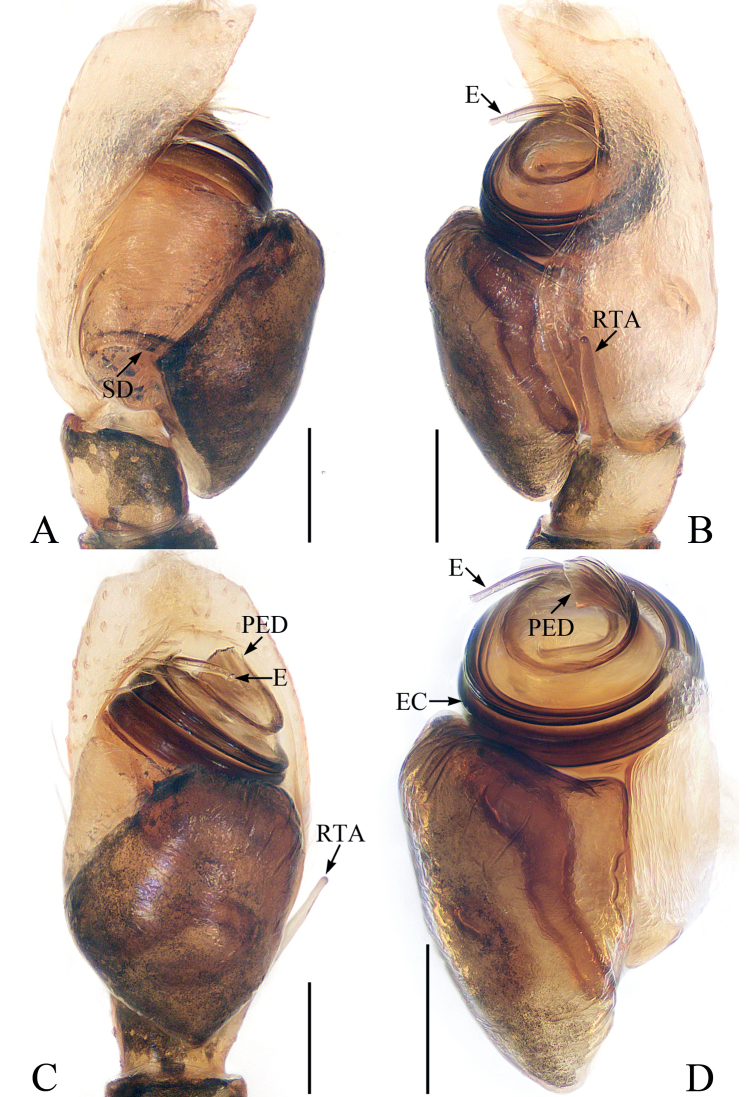
Male palp of *Marengoganae* sp. nov., holotype **A** prolateral **B** retrolateral, **C** ventral **D** bulb, retrolateral. Scale bars: 0.1 mm. Abbreviations: E – embolus; EC – embolic coil; PED – process of embolic disc; RTA – retrolateral tibial apophysis; SD – sperm duct.

**Figure 7.**
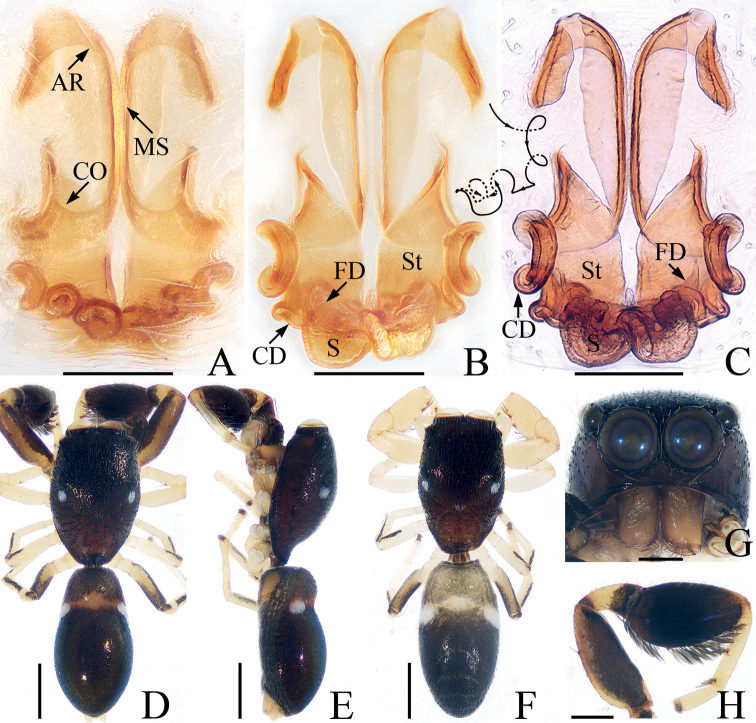
*Marengoganae* sp. nov., male holotype and female paratype **A** epigyne, ventral **B, C** vulva, dorsal **D** holotype habitus, dorsal **E** ditto, lateral **F** female paratype habitus, dorsal **G** holotype carapace, frontal **H** holotype leg I, prolateral. Scale bars: 0.1 mm (**A–C**); 0.2 mm (**G, H**); 0.5 mm (**D–F**). Abbreviations: AR – atrial ridge; CD – copulatory duct; CO – copulatory opening; FD – fertilization duct; MS – median septum; S – spermatheca; St – stiffener.

*Marengoganae* sp. nov. resembles *M.tangi* Wang & Li, 2021 known from Yunnan of China in having a similar habitus and copulatory organs but it can be easily distinguished by the following characters: (1) the process of the embolic disc is lamellar (Fig. 6B, D), whereas it is almost hook-shaped in *M.tangi* (Wang and Li 2021: fig. 6B, D); (2) the RTA is slightly longer than the tibia (Fig. 6B), whereas it is ~ 1/2 the tibial length in *M.tangi* (Wang and Li 2021: fig. 6B); (3) the epigynal stiffener is ~ 4 × wider than the copulatory duct (Fig. 7B, C), whereas it is ~ 2 × wider than copulatory duct in *M.tangi* (Wang and Li 2021: fig. 7B). The species is also similar to that of *M.striatipes* Simon, 1900 known from Sri Lanka, but it can be easily distinguished by elongated atria having U-shaped posterior margins (Fig. 7A), whereas oval atria with C-shaped posterior margins in *M.striatipes* (Benjamin 2004: fig. 67B).

##### Description.

**Male** (Figs 6, 7D, E, G, H). Total length 2.57. Carapace 1.19 long, 0.82 wide. Abdomen 1.35 long, 0.72 wide. Eye sizes and inter-distances: AME 0.28, ALE 0.10, PLE 0.12, AERW 0.71, PERW 0.74, EFL 0.53. Leg measurements: I 2.92 (0.83, 0.43, 0.78, 0.60, 0.28), II 1.85 (0.55, 0.28, 0.43, 0.35, 0.24), III 1.63 (0.48, 0.23, 0.33, 0.35, 0.24), IV 2.16 (0.65, 0.30, 0.53, 0.43, 0.25). Carapace red-brown to dark brown, with pair of white spots of scales behind PLEs, covered with small papillae and brown setae. Chelicerae dark yellow, with two promarginal and three retromarginal teeth. Endites longer than wide. Labium colored as endites. Sternum almost oval. Legs I with enlarged tibiae with dense, ventral, leaf-like scales, and three pairs of ventral macrosetae; other legs pale to brown, with prolateral stripes on femora, patella, and tibiae III, IV. Abdomen elongate-oval, slightly constricted at anterior 1/4, dorsum red-brown to dark brown, anteriorly with a transverse yellow band bearing pair of white spots of scales at lateral margins, covered entirely by large scutum; venter paler than dorsum. Palp (Fig. 6A–D): tibia wider than long; RTA straight, slender, almost 1.5 × longer than tibia, blunt apically; bulb swollen, divided by cleft; embolus coiled almost twice; process of embolic disc lamellar, wrinkled.

**Female** (Fig. 7A–C, F). Total length 2.65. Carapace 1.17 long, 0.79 wide. Abdomen 1.40 long, 0.75 wide. Eye sizes and inter-distances: AME 0.28, ALE 0.10, PLE 0.11, AERW 0.70, PERW 0.73, EFL 0.53. Leg measurements: I 2.17 (0.63, 0.33, 0.55, 0.43, 0.23), II 1.58 (0.48, 0.25, 0.33, 0.30, 0.22), III 1.48 (0.45, 0.23, 0.30, 0.28, 0.22), IV 2.12 (0.66, 0.28, 0.50, 0.43, 0.25). Habitus (Fig. 7F) similar to that of male except paler and with unmodified tibiae I lacking ventral, leaf-like scales. Epigyne (Fig. 7A–C): with pair of anterior hood-shaped structures, and pair of stiffeners touching copulatory ducts; atria elongate-oval, separated by narrow median septum; copulatory openings beneath the posterior margins of atria; copulatory ducts curved, twisted into tortuous coils; spermathecae slightly broadened and curved medially; fertilization ducts lamellar, broad, extending anterolaterally.

##### Distribution.

Only known from the type locality on Hainan Island, China.

#### ﻿ Marengo zhengi sp. nov.

Taxon classificationAnimaliaAraneaeSalticidae

62FA48F1-D0D0-594D-A508-EC44CADFC4B1

https://zoobank.org/5690ACA3-CD79-48CB-86F9-D89B9D95415A

Figs 8 , 9

##### Type material.

***Holotype*** ♂ (IZCAS-Ar43186), China: Hainan: Lingshui County, Diaoluoshan National Nature Reserve, mountain near the river (18°43.39'N, 109°51.27'E, ca. 930 m), 10.viii.2010, G. Zheng leg. ***Paratypes*** 3♂2♀ (IZCAS-Ar43187–43191), same data as holotype; 2♂ (IZCAS-Ar43192–43193), around the Plank Road (18°43.56'N, 109°51.99'E, ca. 950 m), 08.viii.2010, G. Zheng leg.; 1♂ (IZCAS-Ar43194), around the Plank Road (18°43.67'N, 109°51.83'E, ca. 990 m), 09.viii.2010, G. Zheng leg.; 1♀ (IZCAS-Ar43195), around the Plank road near the waterfall (18°43.44'N, 109°52.60'E, ca. 500 m), 10.viii.2010, G. Zheng leg.

##### Etymology.

The specific name is a patronym of Prof. Guo Zheng, the collector of the new species; noun (name) in genitive case.

##### Diagnosis.

**Figure 8.**
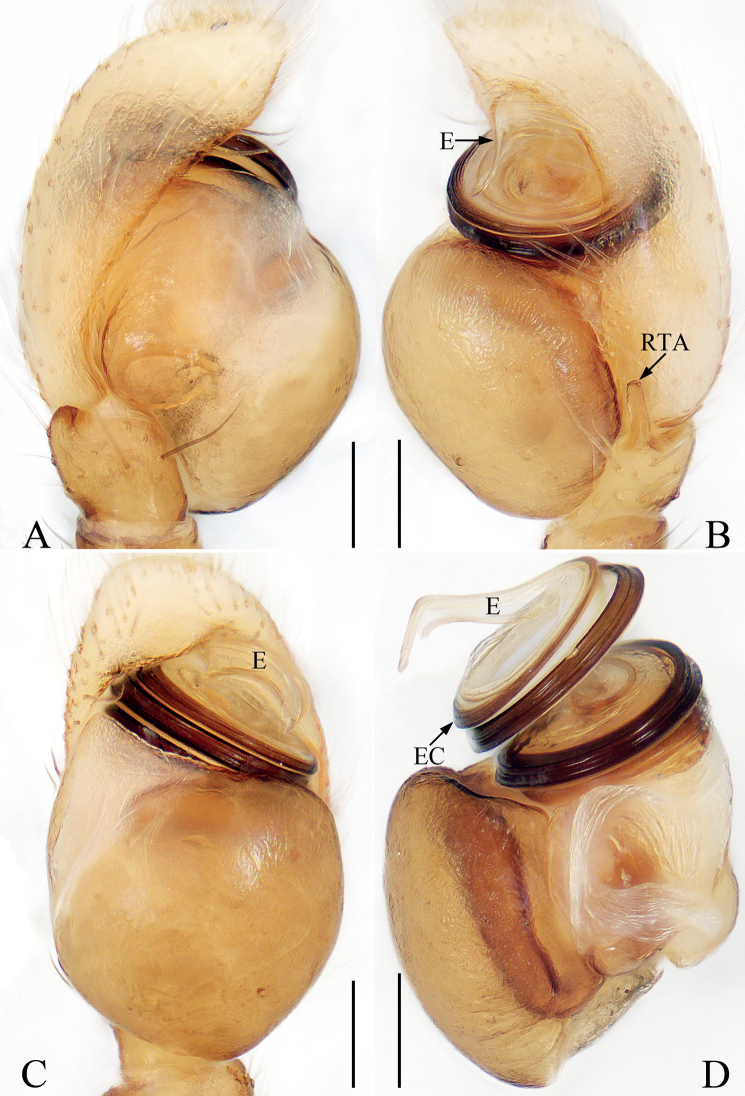
Male palp of *Marengozhengi* sp. nov., holotype and paratype **A** holotype palp, prolateral **B** ditto, retrolateral **C** ditto, ventral **D** paratype bulb, retrolateral. Scale bars: 0.1 mm. Abbreviations: E – embolus; EC – embolic coil; RTA – retrolateral tibial apophysis.

**Figure 9.**
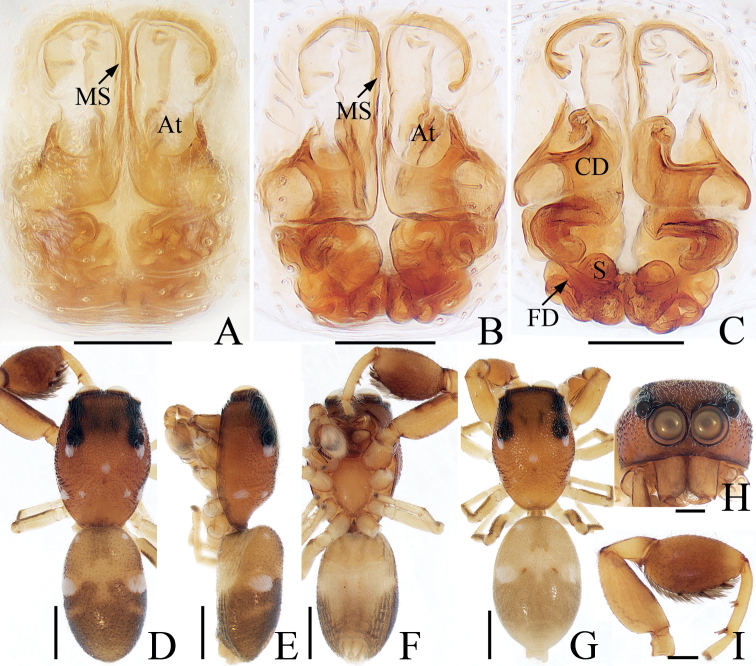
*Marengozhengi* sp. nov., male holotype and female paratype **A, B** epigyne, ventral **C** vulva, dorsal **D** holotype habitus, dorsal **E** ditto, lateral **F** ditto, ventral **G** female paratype habitus, dorsal **H** holotype carapace, frontal **I** holotype leg I, retrolateral. Scale bars: 0.1 mm (**A–C**); 0.2 mm (**H, I**); 0.5 mm (**D–G**). Abbreviations: At – atrium; CD – copulatory duct; FD – fertilization duct; MS – median septum; S – spermatheca.

*Marengozhengi* sp. nov. closely resembles *M.deelemanae* Benjamin, 2004 known from Prachuap Khiri Khan of Thailand in having a similar habitus and copulatory organs, but it differs in the following characters: (1) the embolus is lamellar distally (Fig. 8B, C, D), whereas it is bar-shaped in *M.deelemanae* (Benjamin 2004: fig. 68E, F); (2) the RTA is almost uniform in width (Fig. 8B), whereas it is acutely narrowed medially in *M.deelemanae* (Benjamin 2004: fig. 68E); (3) the copulatory ducts are extending exceed the copulatory openings proximally (Fig. 9C), whereas not exceed the copulatory openings in *M.deelemanae* (Benjamin 2004: fig. 68D).

##### Description.

**Male** (Figs 8, 9D–F, H, I). Total length 2.60. Carapace 1.34 long, 0.96 wide. Abdomen 1.32 long, 0.84 wide. Eye sizes and inter-distances: AME 0.28, ALE 0.10, PLE 0.12, AERW 0.71, PERW 0.75, EFL 0.48. Leg measurements: I 3.14 (0.88, 0.50, 0.83, 0.68, 0.25), II 1.81 (0.58, 0.30, 0.40, 0.33, 0.20), III 1.71 (0.53, 0.25, 0.35, 0.38, 0.20), IV 2.11 (0.68, 0.33, 0.50, 0.40, 0.20). Carapace elongate-oval, red-yellow, with a pair of dark dots on eye field and seven clusters of white patches of scales on thorax, covered with small papillae and thin setae. Chelicerae dark yellow, with two promarginal and four retromarginal teeth. Endites colored as chelicerae, with dense, dark setae anteriorly. Labium darker than endites, almost linguiform. Sternum almost oval. Legs yellow to red-yellow; leg I robust, with enlarged tibia with dense, leaf-like scales, and three pairs of macrosetae ventrally; other legs pale to brown. Abdomen elongate-oval, dorsum yellow to dark brown, with pair of small, round, white spots of scales at anterolateral margin, a broad, longitudinal, brown band followed by irregular yellow patch, and pair of round, white patches mediolaterally, entirely covered by large scutum; venter pale to brown. Palp (Fig. 8A–D): tibia short, with straight RTA of nearly uniform width, slightly shorter than its length, and blunt apically in retrolateral view; bulb bugling; embolus coiled ~3 ×, tapered, lamellar distally.

**Female** (Fig. 9A–C, G). Total length 2.73. Carapace 1.23 long, 0.87 wide. Abdomen 1.40 long, 0.92 wide. Eye sizes and inter-distances: AME 0.29, ALE 0.10, PLE 0.12, AERW 0.70, PERW 0.77, EFL 0.48. Leg measurements: I 2.14 (0.65, 0.33, 0.53, 0.40, 0.23), II 1.61 (0.50, 0.25, 0.33, 0.30, 0.23), III 1.59 (0.45, 0.23, 0.30, 0.38, 0.23), IV 1.96 (0.63, 0.25, 0.45, 0.40, 0.23). Habitus (Fig. 9G) similar to that of male except pale, with unmodified tibia I lacking leaf-like scales and replaced with several long setae ventrally. Epigyne (Fig. 9A–C): atria paired, anteriorly located, ~ 1.5 × longer than wide, separated by narrow septum; copulatory openings beneath posterior edges of atria; copulatory ducts long, widened proximally, forming complicated coils; spermathecae sub-oval; fertilization ducts elongated, lamellar.

##### Distribution.

Only known from the type locality on Hainan Island, China.

#### ﻿Genus Nungia

Taxon classificationAnimaliaAraneaeSalticidae

Żabka, 1985

A64BE397-906B-5359-BD18-CC880E4846EA

##### Type species.

*Nungiaepigynalis* Żabka, 1985 from Vietnam by original designation.

##### Comments.

*Nungia* contains five species distributed mainly in Southeast Asia (WSC 2022). The original definition of the genus is just based on the butterfly-shaped vulva (Żabka 1985). Moreover, Maddison et al. (2020) transferred four species into the genus based on molecular evidence and the transferred species are inconsistent in copulatory organs. Based on that, it is hard to define the genus according to its morphological character at present.

#### ﻿ Nungia tangi sp. nov.

Taxon classificationAnimaliaAraneaeSalticidae

910D6C6D-4C32-5F9F-9224-9AF0D142DB7E

https://zoobank.org/85DDE8DF-DE75-4859-9E95-8335506E73AA

Figs 10 , 11

##### Type material.

***Holotype*** ♂ (TRU-JS 0634), China: Hainan: Ledong County, Jianfeng Village, Jianfengling National Nature Reserve, Yulingu (18°44.96'N, 108°55.32'E, ca. 650 m), 13.iv.2020, C. Wang & Y.F. Yang leg. ***Paratype***: 1♀ (TRU-JS 0635), same data as holotype.

##### Etymology.

The specific name is a patronym of Dr. Guo Tang, who conducted important research on the taxonomy of the crab spiders of Hainan Island; noun (name) in genitive case.

##### Diagnosis.

**Figure 10.**
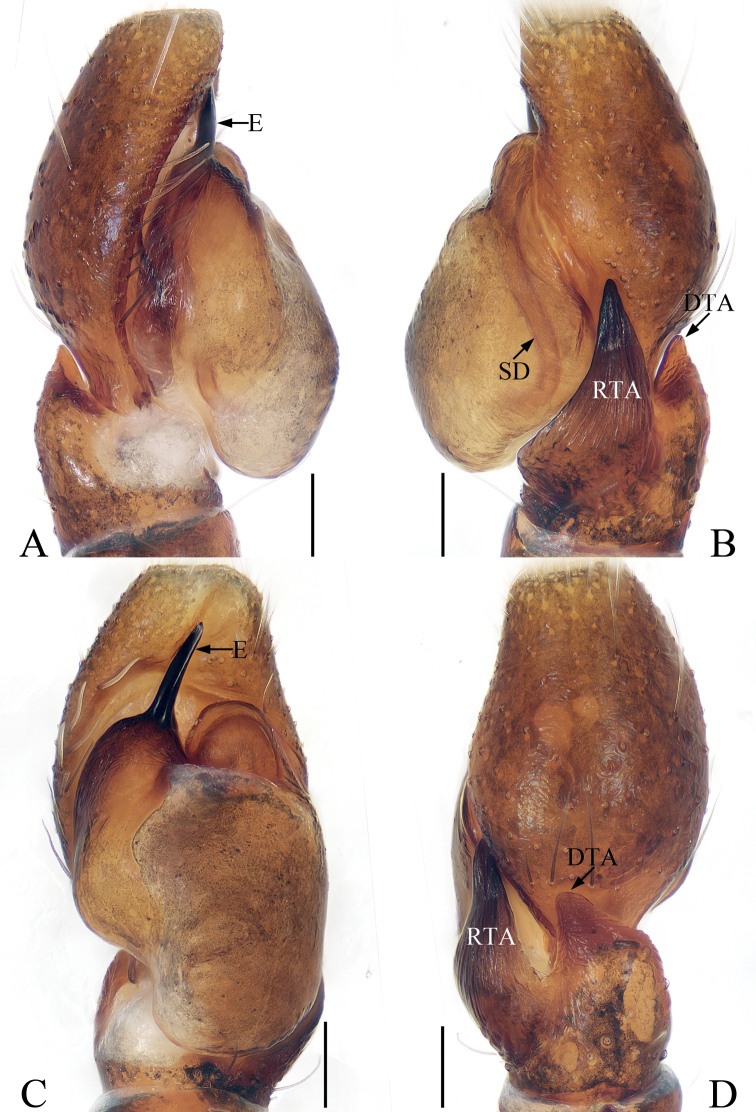
Male palp of *Nungiatangi* sp. nov., holotype **A** prolateral **B** retrolateral **C** ventral **D** dorsal. Scale bars: 0.1 mm. Abbreviations: DTA – dorsal tibial apophysis; E – embolus; RTA – retrolateral tibial apophysis; SD – sperm duct.

**Figure 11.**
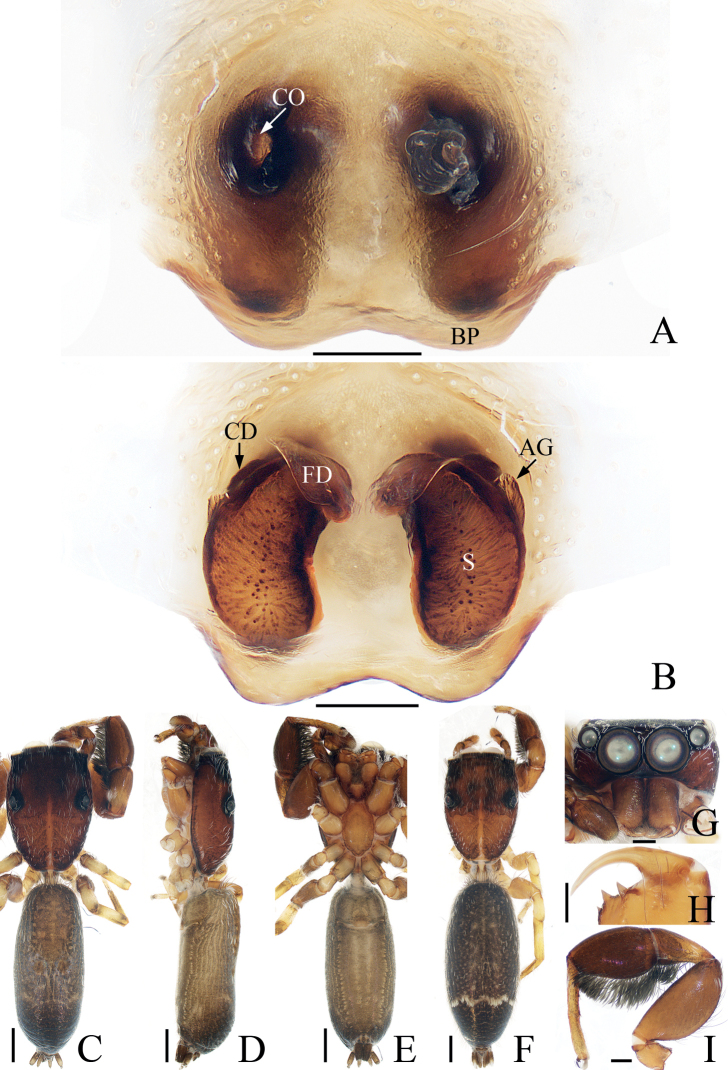
*Nungiatangi* sp. nov., male holotype and female paratype **A** epigyne, ventral **B** vulva, dorsal **C** holotype habitus, dorsal **D** ditto, lateral **E** ditto, ventral **F** female paratype habitus, dorsal **G** holotype carapace, frontal **H** holotype chelicera, posterior **I** holotype leg I, prolateral. Scale bars: 0.1 mm (**A, B, H**); 0.2 mm (**G, I**); 0.5 mm (**C–F**). Abbreviations: AG – accessory gland; BP – basal epigynal plate; CD – copulatory duct; CO – copulatory opening; FD – fertilization duct; S – spermatheca.

*Nungiatangi* sp. nov. resembles *N.epigynalis* Żabka, 1985 known from China, Vietnam, and Japan in the general shape of copulatory organs, but it can be easily distinguished by the following characters: (1) the presence of DTA (Fig. 10B, D), whereas absent in *N.epigynalis* (Peng 2020: fig. 184f); (2) the RTA is directed upward in retrolateral view (Fig. 10B), whereas it is curved retrolaterally in *N.epigynalis* (Peng 2020: fig. 184f); (3) the spermathecae are eggplant-shaped (Fig. 11B), whereas they are oval in *N.epigynalis* (Peng 2020: fig. 184 i, j).

##### Description.

**Male** (Figs 10, 11C–E, G–I). Total length 4.63. Carapace 1.83 long, 1.33 wide. Abdomen 2.63 long, 1.04 wide. Eye sizes and inter-distances: AME 0.41, ALE 0.20, PLE 0.20, AERW 1.21, PERW 1.29, EFL 0.83. Leg measurements: I 3.92 (1.13, 0.78, 0.88, 0.63, 0.50), II 2.58 (0.78, 0.50, 0.60, 0.40, 0.30), III 2.43 (0.75, 0.40, 0.45, 0.53, 0.30), IV 3.25 (1.05, 0.50, 0.75, 0.65, 0.30). Carapace red-brown, with an irregular dark patch in eye field and narrow, orange central stripe posteromedially, covered with sparse, white setae. Fovea punctiform. Chelicerae dark yellow, with two promarginal teeth and one retromarginal tooth. Endites colored as chelicerae, longer than wide, slightly widened distally, with dense setae on inner margins. Labium sub-linguiform, paler terminally. Sternum elongate-oval, > 1.5 × longer than wide. Legs I robust, with slightly enlarged femora and tibiae, and bearing dense, leaf-like scales ventrally on patellae and tibiae; other legs yellow to brown. Abdomen elongated, dorsum brown to dark brown, with the transverse, undulate streak at posterior 1/3, partly covered by a scutum anteromedially; venter pale to brown, with lateral, dotted lines. Palp (Fig. 10A–D): tibia short, with tapered RTA strongly sclerotized at distal 1/3, and blunt apically, and sub-triangular DTA; bulb swollen; embolus straight, strongly sclerotized, originates from antero-prolateral edge of bulb, extending antero-retrolaterally, with blunt apex.

**Female** (Fig. 11A, B, F). Total length 5.75. Carapace 2.04 long, 1.51 wide. Abdomen 3.23 long, 1.45 wide. Eye sizes and inter-distances: AME 0.43, ALE 0.22, PLE 0.22, AERW 1.30, PERW 1.43, EFL 0.89. Leg measurements: I 3.02 (0.78, 0.65, 0.78, 0.43, 0.38), II missing, III 2.65 (0.75, 0.45, 0.50, 0.65, 0.30), IV 3.66 (1.13, 0.63, 0.90, 0.70, 0.30). Habitus (Fig. 11F) similar to that of male except without dorsal abdominal scutum. Epigyne (Fig. 11A, B): almost as long as wide, with arc-shaped basal plate; copulatory openings almost round, anteriorly located, separated from each other by ~ 1/2 width of basal plate; copulatory ducts very short, with lamellar accessory glands; spermathecae eggplant-shaped, separated from each other by 1/3 their width; fertilization ducts lamellar, broad, extending anterolaterally.

##### Distribution.

Only known from the type locality, Hainan Island, China.

##### Comments.

The species is placed into *Nungia* due to its general resemblance to the *N.epigynalis* Żabka, 1985. However, it also possesses some characters, such as the presence of long, dense, leaf-like scales ventrally on the patellae and tibiae I in both sexes, having two tibia apophyses of male palp and elongated spermathecae which are different from the latter. And so, its generic position may need further confirmation.

#### ﻿Genus Pengmarengo gen. nov.

Taxon classificationAnimaliaAraneaeSalticidae

FF1249D6-784D-584E-A356-3C77CDC3E7F6

https://zoobank.org/BA4E5B47-123C-4918-889C-F0C39EC9FF26

##### Type species.

*Pengmarengoyangi* sp. nov. from China.

##### Etymology.

The generic name is a combination of the first name of Prof. Xianjin Peng, a renowned jumping spider expert, and the related genus *Marengo*. The gender is feminine.

##### Diagnosis.

*Pengmarengo* gen. nov. can be easily distinguished from Ballini genera except for *Afromarengo*, *Indomarengo*, *Leikung*, and *Marengo* by the presence of ventral, leaf-like scales on tibiae I (Benjamin 2004; Azarkina and Haddad 2020). It can be easily distinguished from *Leikung* by the not raised PME and only five macrosetae on tibiae I, whereas PME is raised and there are eight macrosetae on tibiae I in *Leikung* (Benjamin 2004: fig. 49D). It differs from the other three genera by the: (1) unmodified femora I, whereas enlarged in *Afromarengo*, and *Marengo* (Azarkina and Haddad 2020: figs 90, 91, 97–99, 105, 106, 112, 113; Wanless 1978: figs 1C, 3E, 10E); (2) very flat carapace, which is > 3 × longer than wide in lateral view, with the facial length almost equal to the AME diameter, and without a distinct clypeus, whereas carapace is < 3 × longer than wide in lateral view, with facial length greater than the AME diameter and with a distinct clypeus in the three genera (Wanless 1978: figs 1D, 3D; Benjamin 2004: figs 38C, 39A; Azarkina and Haddad 2020: figs 88, 89, 94, 96, 103, 104, 110, 111, 121, 122, 142, 143); (3) the presence of pair of white patches on the dorsum of abdomen, whereas absent in *Afromarengo*, and *Indomarengo* (Benjamin 2004: figs 38A, 41C, 42E; Azarkina and Haddad 2020: figs 87, 92 100, 108, 119, 142); (4) specific form of the copulatory ducts which extend posterolaterally before reversing direction completely or partly, causing the copulatory ducts to overlap anteromedially, and the prominent spermathecae, whereas copulatory ducts do not overlap and spermathecae are not prominent in the three genera (Wanless 1978: figs 1J, 3C; Benjamin 2004: fig. 39C; Azarkina and Haddad 2020: figs 83, 138).

##### Description.

Small to medium spiders, both sexes with similar habitus. Carapace flat, > 3 × longer than wide in lateral view, covered with small papillae and larger piliferous papillae, usually with four clusters of white scales, of which two postero-lateral to AMEs and other two posterolaterally located on thorax. Fovea and clypeus indistinct. Chelicerae yellow to red-brown, with two promarginal and three retromarginal teeth. Endites longer than wide, with pale ental sides bearing dark setae. Labium usual shape. Sternum elongated, sub-fusiform. Legs I robust, with enlarged tibia with a cluster of ventral, leaf-like scales and five ventral macrosetae in both sexes, other legs pale to yellow, mostly with dark brown stripes laterally on femora and tibiae. Abdomen elongated, > 2.5 × longer than wide, slightly constricted at anterior 1/3 in males, dorsum with pair of white patches of setae laterally behind constriction, entirely covered by scutum in males, and with sub-trapeziform scutum near anterior margin in females.

Palp: tibia wider than long, with bar-shaped RTA of varying lengths; bulb bulging, divided by a cleft; embolus short, coiled < 2 ×, associated with disc process or not. Epigyne: longer than wide; atria paired, oval, with arc-shaped anterior ridge; copulatory ducts long, extending posterolaterally before reversing direction completely or partly, causing ducts to overlap anteromedially; spermathecae prominent, L- or U-shaped, with or without hemispherical processes at anterior margins; fertilization ducts originating from the median or anterior portions of longitudinal parts of spermathecae.

##### Distribution.

China (Hainan, Yunnan, Taiwan), Indonesia.

##### Composition.

*Pengmarengo* is a tribe Ballini genus, and currently includes five species: *P.chelifer* (Simon, 1900), comb. nov. (transferred from *Philates*), *P.elongata* (Peng & Li, 2002), comb. nov. (transferred from *Tauala*), *P.wengnan* (Wang & Li, 2022), comb. nov. (transferred from *Indomarengo*, see Wang and Li 2022), *P.yangi* sp. nov., and *P.yui* (Wang & Li, 2020), comb. nov. (transferred from *Indomarengo*).

##### Comments.

*P.yui* and *P.wengnan* are transferred because they are sharing similar habitus and copulatory organs with generotype, especially in having the partly overlapped copulatory ducts and prominent, L-shaped spermathecae. *P.chelifer* possesses a series of characters, such as the presence of ventral scales of tibial I (rather than ventral setae in *Philates*), with sub-trapeziform scutum on the dorsum of abdomen in female, the flat carapace, pair of white patches of setae laterally on the dorsum of abdomen, and anteromedially overlapped copulatory ducts (Benjamin 2004: figs 25D, 26B, C, 27A–C), which are consistent with the generotype, and so, it also being transferred. According to the diagnostic drawings (Peng 2020: fig. 344), *P.elongata* is a Ballini species. Moreover, it has very flat carapace and anteromedially overlapped copulatory ducts. Based on that, we also transferred it into the new genus.

#### ﻿ Pengmarengo yangi sp. nov.

Taxon classificationAnimaliaAraneaeSalticidae

84389095-FC6E-5F8E-A069-2AA246AC8770

https://zoobank.org/1878BD48-9161-444B-8DA8-8FF3520528BB

Figs 12 , 13 , 14

##### Type material.

***Holotype*** ♂ (TRU-JS 0636), China: Hainan: Ledong County, Jianfeng Township, Jianfengling National Nature Reserve, Main Peak (18°43.11'N, 108°52.32'E, ca. 1400 m), 16.v.2019, C. Wang & Y.F. Yang leg. ***Paratypes*** 2♂3♀ (TRU-JS 0637–0641), same data as holotype.

##### Etymology.

The specific name is a patronym of Mr. Yuanfa Yang, one of the collectors of the type specimens; noun (name) in genitive case.

##### Diagnosis.

**Figure 12.**
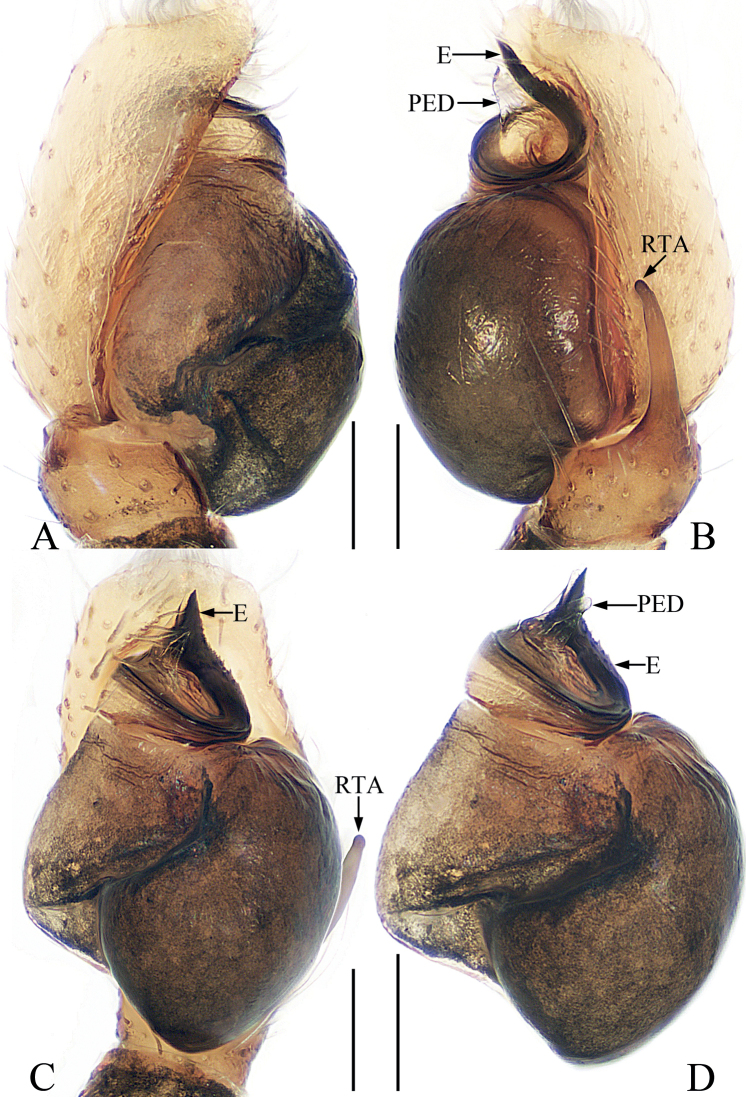
Male palp of *Pengmarengoyangi* sp. nov., holotype **A** prolateral **B** retrolateral **C** ventral **D** bulb, ventral. Scale bars: 0.1 mm. Abbreviations: E – embolus; PED – process of embolic disc; RTA – retrolateral tibial apophysis.

The male of *Pengmarengoyangi* sp. nov. resembles *P.yui* (Wang & Li, 2020) comb. nov. known from Yunnan of China by the habitus, but it can be easily distinguished by the lamellar process of the embolic disc and the flat embolus has spine-shaped distal processes (Figs 12B–D, 14B), whereas lacks the process of embolic disc and with bar-shaped embolus lacks process in *P.yui* (Wang and Li 2020: figs 5B–D, 6A, B). The female can be easily distinguished from other congeners by the prominent, L-shaped spermathecae, whereas in *P.chelifer* and *P.elongata* they are U-shaped or elongated (Benjamin 2004: fig. 26C; Peng 2020: fig. 344c).

##### Description.

**Male** (Figs 12, 14A–C, E–G). Total length 3.07. Carapace 1.36 long, 0.82 wide. Abdomen 1.72 long, 0.64 wide. Eye sizes and inter-distances: AME 0.28, ALE 0.12, PLE 0.11, AERW 0.75, PERW 0.78, EFL 0.51. Leg measurements: I 3.08 (0.80, 0.45, 0.93, 0.70, 0.20), II 1.68 (0.50, 0.25, 0.38, 0.33, 0.20), III 1.55 (0.43, 0.23, 0.33, 0.33, 0.23), IV 2.01 (0.60, 0.28, 0.50, 0.40, 0.23). Carapace red-brown to dark brown, with two clusters of white setae near PLEs and two posteriorly, covered with small papillae and thin setae. Chelicerae with two promarginal teeth and three retromarginal teeth fused basally. Legs I robust, with enlarged tibiae with dense, ventral, leaf-like scales, two prolatero-ventral and three retrolatero-ventral tibial macrosetae and two pairs of ventral metatarsal macrosetae, respectively; remaining legs yellow to pale yellow, with brown stripes on femora. Abdomen elongated, slightly constricted at anterior 1/3, dorsum brown to dark brown, with pair of oval white patches of setae on lateral margins behind constriction, entirely covered by large scutum and thin setae; venter dark brown. Palp (Fig. 12A–D): tibia wider than long; RTA ~ 1.6 × longer than tibia, slightly curved distally to a blunt tip; bulb swollen, divided by pale, oblique cleft; embolus short, coiled ca. a circle, pointed apically, with spine-shaped processes distally; process of embolic disc lamellar, membranous, with two parts.

**Figure 13.**
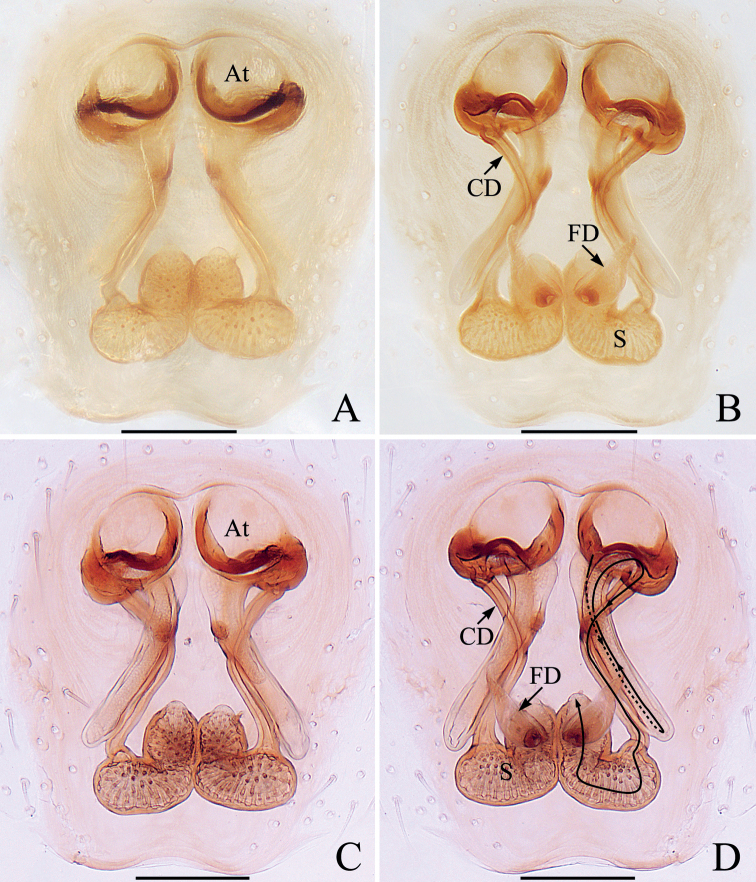
Epigyne-vulva of *Pengmarengoyangi* sp. nov., female paratype **A, C** epigyne, ventral **B, D** vulva, dorsal. Scale bars: 0.1 mm. Abbreviations: At – atrium; CD – copulatory duct; FD – fertilization duct; S – spermatheca.

**Figure 14.**
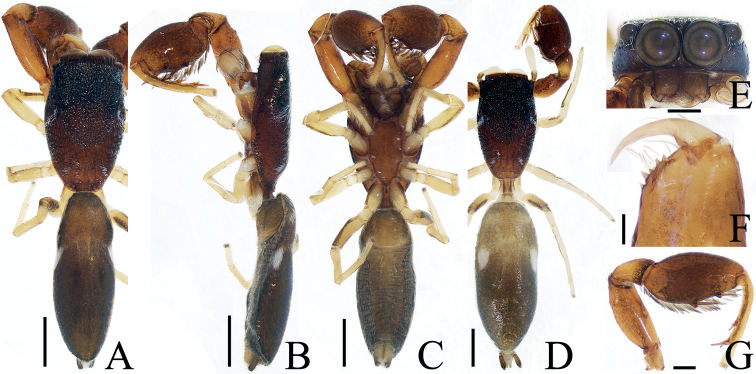
*Pengmarengoyangi* sp. nov., male holotype and female paratype **A** holotype habitus, dorsal **B** ditto, lateral **C** ditto, ventral **D** female paratype habitus, dorsal **E** holotype carapace, frontal **F** holotype chelicera, posterior **G** holotype leg I, prolateral. Scale bars: 0.1 mm (**F**); 0.2 mm (**E, G**); 0.5 mm (**A–D**).

**Female** (Figs 13, 14D). Total length 3.67. Carapace 1.33 long, 0.80 wide. Abdomen 2.02 long, 0.84 wide. Eye sizes and inter-distances: AME 0.29, ALE 0.11, PLE 0.11, AERW 0.75, PERW 0.78, EFL 0.51. Leg measurements: I 2.39 (0.63, 0.35, 0.70, 0.53, 0.18), II 1.59 (0.50, 0.25, 0.38, 0.28, 0.18), III 1.53 (0.45, 0.23, 0.35, 0.30, 0.20), IV 2.01 (0.63, 0.28, 0.50, 0.40, 0.20). Habitus (Fig. 14D) similar to that of male except lacks abdominal scutum, constriction, but with sub-trapeziform scutum near anterior margin. Epigyne (Fig. 13A–D): longer than wide, with pair of round atria anteriorly; copulatory openings beneath the posterior margins of atria; copulatory ducts long, posterolaterally extending before reversing direction at proximal 2/3, and followed by the S-shaped thinner portions connected to anterolateral edges of spermathecae; spermathecae L-shaped, touching; fertilization ducts originate from the center of longitudinal extended parts of spermathecae, extending anterolaterally.

##### Distribution.

Only known from the type locality on Hainan Island, China.

#### ﻿Genus Philates

Taxon classificationAnimaliaAraneaeSalticidae

Simon, 1900

1F62E83E-D2F3-5EAF-B0E4-DDACF24B1171

##### Type species.

*Philatesgrammicus* Simon, 1900 from Philippines by original designation.

##### Comments.

The genus *Philates* is belonging to the tribe Ballini and is represented by ten species distributed from Southeast Asia to Papua New Guinea (Maddison 2015; WSC 2022). A recent re-defined of the genus was also provided by Benjamin (2004), who diagnosed the genus by the absence of carapace protuberance, and the presence of leaf-like setae ventrally on tibiae I. However, certainly, the genus definition is too broad and that has also been noted by Benjamin (2004), who mentioned the Papuan New Guinea species could be divided into another genus. Herein, a proper definition of the genus is not discussed, and we placed *Philateszhoui* sp. nov. into the genus due to it possesses the leaf-like setae ventrally on tibiae I and shares a similar carapace shape with the generotype.

#### ﻿ Philates zhoui sp. nov.

Taxon classificationAnimaliaAraneaeSalticidae

F005EE79-0542-5350-9E00-AAE92AE75125

https://zoobank.org/54C7065F-031A-49BA-9CAE-C252FEC9DB1D

Figs 15 , 16

##### Type material.

***Holotype*** ♂ (IZCAS-Ar43206), China: Hainan: Baisha County, Yinggeling National Nature Reserve (19°02.93'N, 109°33.65'E, ca. 730 m), 20.viii.2010, G. Zheng leg. ***Paratypes*** 20♂17♀ (IZCAS-Ar43207–43243), same data as holotype.

##### Etymology.

The specific name is a patronym of Mr. Runbang Zhou, our guide in Jianfengling National Nature Reserve; noun (name) in genitive case.

##### Diagnosis.

**Figure 15.**
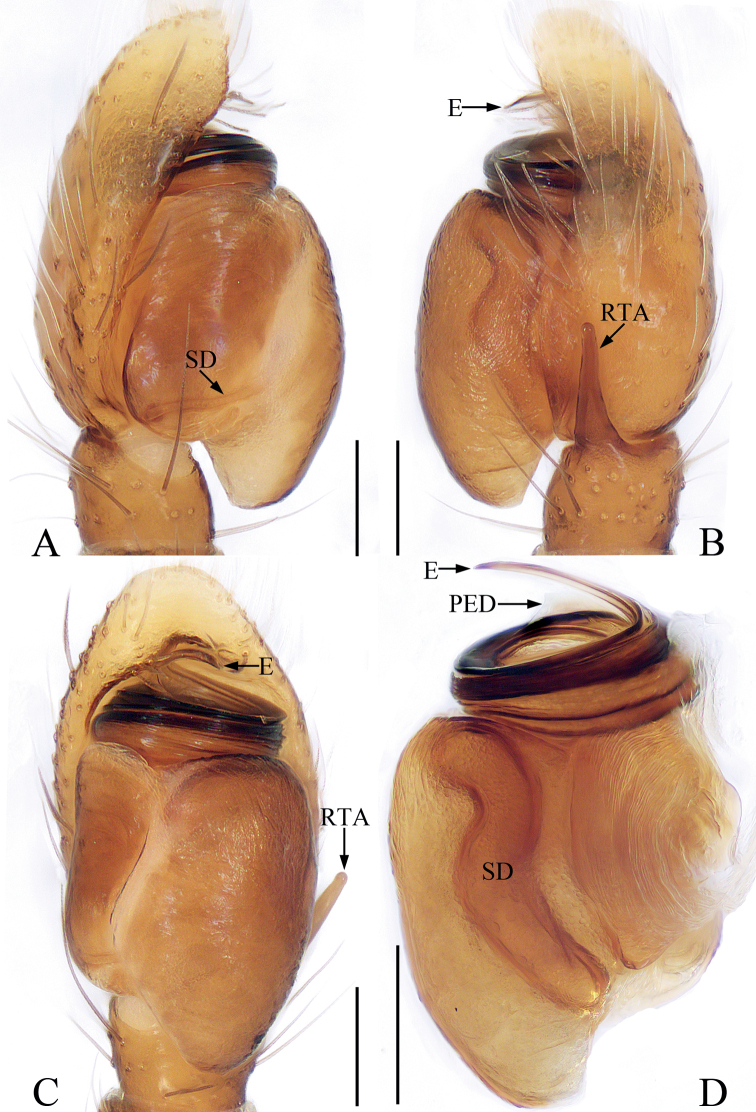
Male palp of *Philateszhoui* sp. nov., holotype **A** prolateral **B** retrolateral, **C** ventral **D** bulb, retrolateral. Scale bars: 0.1 mm. Abbreviations: E – embolus; PED – process of embolic disc; RTA – retrolateral tibial apophysis; SD – sperm duct.

**Figure 16.**
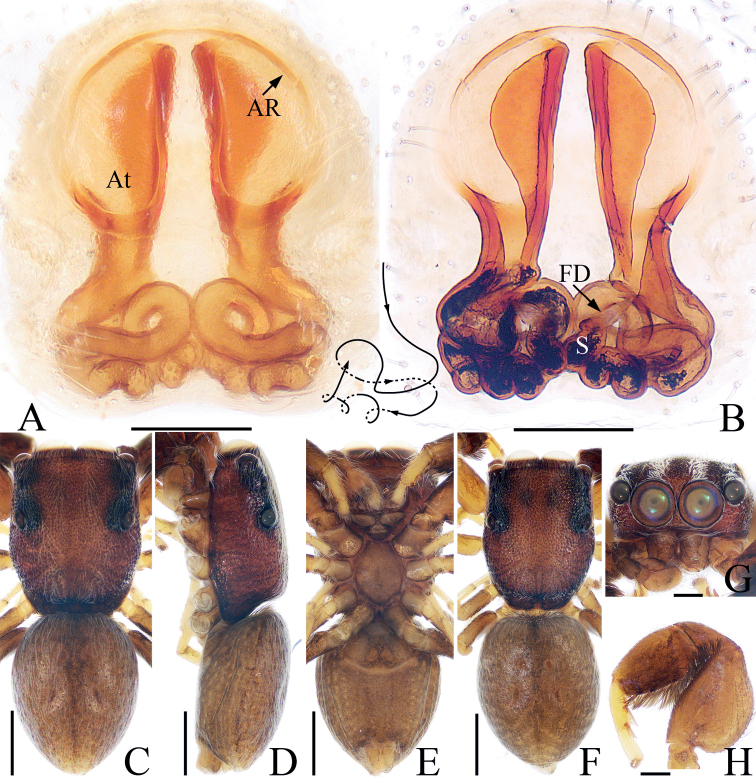
*Philateszhoui* sp. nov., male holotype and female paratype **A** epigyne, ventral **B** vulva, dorsal **C** holotype habitus, dorsal **D** ditto, lateral **E** ditto, ventral **F** female paratype habitus, dorsal **G** holotype carapace, frontal **H** holotype leg I, prolateral. Scale bars: 0.1 mm (**A, B**); 0.2 mm (**G, H**); 0.5 mm (**C–F**). Abbreviations: AR – atrial ridge; At – atrium; FD – fertilization duct; S – spermatheca.

*Philateszhoui* sp. nov. resembles that of *P.grammicus* Simon, 1900 known from Philippines and Indonesia in the carapace sloping steeply at the posterior sub-margin, the enlarged femora I, and the dense setae ventrally on the enlarged tibia I, but it can be easily distinguished by the following characters: (1) the RTA is longer than the tibia (Fig. 15B), whereas it is ~ 1/2 the tibia length in *P.grammicus* (Wanless 1978: fig. 10J); (2) the copulatory ducts are separated from each other proximally by their width (Fig. 16A, B), whereas they are touching in *P.grammicus* (Wanless 1978: fig. 10D, H); (3) the male carapace lacks patches of scales (Fig. 16C, D), whereas there are patches of white scales behind the PMEs and on the slope of the thorax in *P.grammicus* (Wanless 1978: fig. 10D, H). The new species is also similar to *Colaxessazailus* Paul, Prajapati, Joseph & Sebastian, 2020 known from India in having very similar copulatory organs, but it can be easily distinguished by the square cephalic region, which is trapeziform in *C.sazailus* (Paul et al. 2020: fig. 6, 15).

##### Description.

**Male** (Figs 15, 16C–E, G, H). Total length 2.51. Carapace 1.30 long, 1.04 wide. Abdomen 1.28 long, 1.02 wide. Eye sizes and inter-distances: AME 0.33, ALE 0.19, PLE 0.15, AERW 0.98, PERW 0.98, EFL 0.57. Leg measurements: I 2.26 (0.75, 0.43, 0.55, 0.33, 0.20), II 1.61 (0.53, 0.25, 0.30, 0.33, 0.20), III 1.56 (0.50, 0.23, 0.30, 0.33, 0.20), IV 1.86 (0.63, 0.25, 0.40, 0.38, 0.20). Carapace red-brown, acutely sloped at posterior sub-margin, the square cephalic region with two longitudinal white streaks of setae medially. Chelicerae with two promarginal and four retromarginal teeth. Endites colored as chelicerae, broadened distally. Labium sub-linguiform, dark brown, paler apically. Sternum yellow-brown, slightly longer than wide. Legs I robust, with enlarged femora and tibiae, and dense, long, dark setae ventrally on femora, patellae and tibiae; other legs pale yellow to dark brown. Abdomen oval, dorsum red-brown, paler at terminus, with longitudinal, irregular dark streak centrally, two pairs of muscle depressions medially, covered entirely by scutum, with dense, thin setae; venter colored as dorsum. Palp (Fig. 15A–D): tibia slightly wider than long, with straight, tapered RTA ~ 1.5 × of its length, and blunt apically; bulb swollen, longer than wide, sperm duct sinuous retrolaterally; embolus coiled ~ 2 ×, pointed at terminus; process of embolus disc lamellar.

**Female** (Fig. 16A, B, F). Total length 2.44. Carapace 1.17 long, 0.92 wide. Abdomen 1.23 long, 1.01 wide. Eye sizes and inter-distances: AME 0.31, ALE 0.16, PLE 0.15, AERW 0.87, PERW 0.87, EFL 0.51. Leg measurements: I 1.76 (0.58, 0.33, 0.40, 0.25, 0.20), II 1.41 (0.43, 0.25, 0.28, 0.25, 0.20), III 1.39 (0.43, 0.23, 0.28, 0.25, 0.20), IV 1.74 (0.58, 0.28, 0.38, 0.30, 0.20). Habitus (Fig. 16F) similar to that of male except with pair of dark spots medially in eye field. Epigyne (Fig. 16A, B): longer than wide; anteriorly located, oval atrium separated by broad, sub-oblong septum ~ 2 × longer than wide; copulatory openings beneath the lowest margin of atrium; copulatory ducts separated, widened proximally, with complex coils; spermathecae elongated; fertilization ducts lamellar, extending anterolaterally.

##### Distribution.

Only known from the type locality in Hainan Island, China.

#### ﻿Genus Toxeus

Taxon classificationAnimaliaAraneaeSalticidae

C.L. Koch, 1846

D5139C3E-84E3-5D3E-8737-BCFD2C4E27DB

##### Type species.

*Toxeusmaxillosus* C. L. Koch, 1846 from Indonesia by original designation.

##### Comments.

*Toxeus* is a Myrmarachnina genus and represented 12 species distributed from East to Southeast Asia (Maddison 2015; WSC 2022). The genus has always been considered a synonym of the genus *Myrmarachne* until was reinstated by Prószyński (2016), who separates nine genera from *Myrmarachne* based on the study of the morphology of copulatory organs and diagnosed *Toxeus* by the pipe-like sclerotized spermathecae. However, Prószyński’s conclusion was denied by the molecular evidence (Yamasaki et al. 2018; Maddison and Szűts 2019) and so the validity of the genus is uncertain. Herein, we assigned the following new species to the genus due to its being morphologically similar to the known species of the genus.

#### ﻿ Toxeus hainan sp. nov.

Taxon classificationAnimaliaAraneaeSalticidae

D767B786-F8FE-5A12-BA01-BEA56BA7AED1

https://zoobank.org/7F0388AD-9659-4ABE-9EE6-542CBC555F32

Figs 17 , 18

##### Type material.

***Holotype*** ♀ (IZCAS-Ar43196), China: Hainan: Lingshui County, Diaoluoshan National Nature Reserve (18°43.39'N, 109°51.27'E, ca. 930 m), 10.viii.2010, G. Zheng leg. ***Paratypes*** 1♂ (IZCAS-Ar43197), same data as holotype; 1♂ (IZCAS-Ar43198), Ledong County, Jianfengling National Nature Reserve, eastern ravine of Mingfenggu (18°64.69'N, 108°51.59'E, ca. 810 m), 17.xii.2007 morning, S. Li leg.; 1♀ (IZCAS-Ar43199), same locality and collector, 18.xii.2007 morning, S. Li leg.; 1♂ (IZCAS-Ar43200), Changjiang County, Bawangling National Nature Reserve, Dong’er Management Station (19°05.75'N, 109°10.56'E, ca. 830 m), 24.xii.2007, S. Li leg.; 1♂ (IZCAS-Ar43201), 5 km ahead of Dong’er Management Station (19°05.19'N, 109°11.80'E, ca. 1010 m), 25.xii.2007, S. Li leg.; 1♀ (IZCAS-Ar43202), Wangxia Village, 26.xii.2007, S. Li leg.; 1♀ (IZCAS-Ar43203), Qicha Township (19°01.95'N, 109°06.15'E, ca. 700 m), 29.xii.2007, S. Li leg.; 1♀ (IZCAS-Ar43204), Dong’er Management Station (19°05.75'N, 109°10.56'E, ca. 830 m), 30.xii.2007, S. Li leg.; 1♀ (IZCAS-Ar43205), Qiongzhong County, Limushan National Nature Reserve (19°11.98'N, 109°43.76'E, ca. 580 m), 13.viii.2007, S. Li leg.

##### Etymology.

The specific name comes from the type locality, Hainan Island; noun in apposition.

##### Diagnosis.

**Figure 17.**
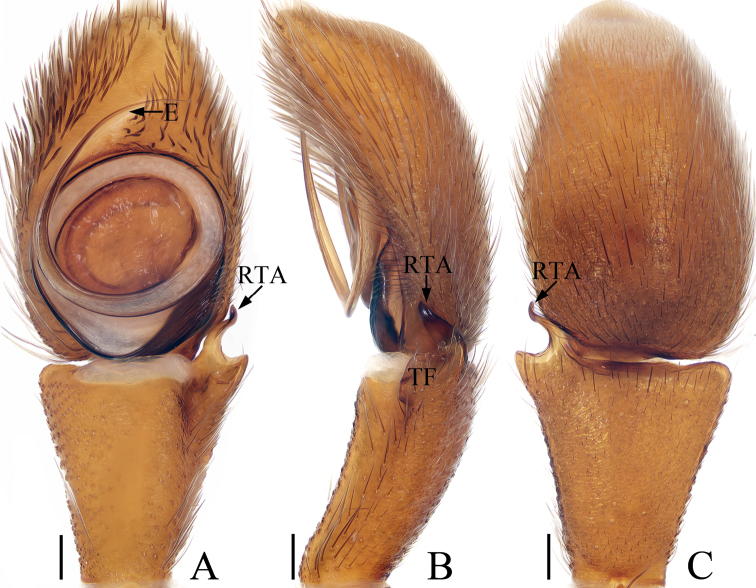
Male palp of *Toxeushainan* sp. nov., paratype **A** ventral **B** retrolateral **C** dorsal. Scale bars: 0.1 mm. Abbreviations: E – embolus; RTA – retrolateral tibial apophysis; TF – tibial flange.

**Figure 18.**
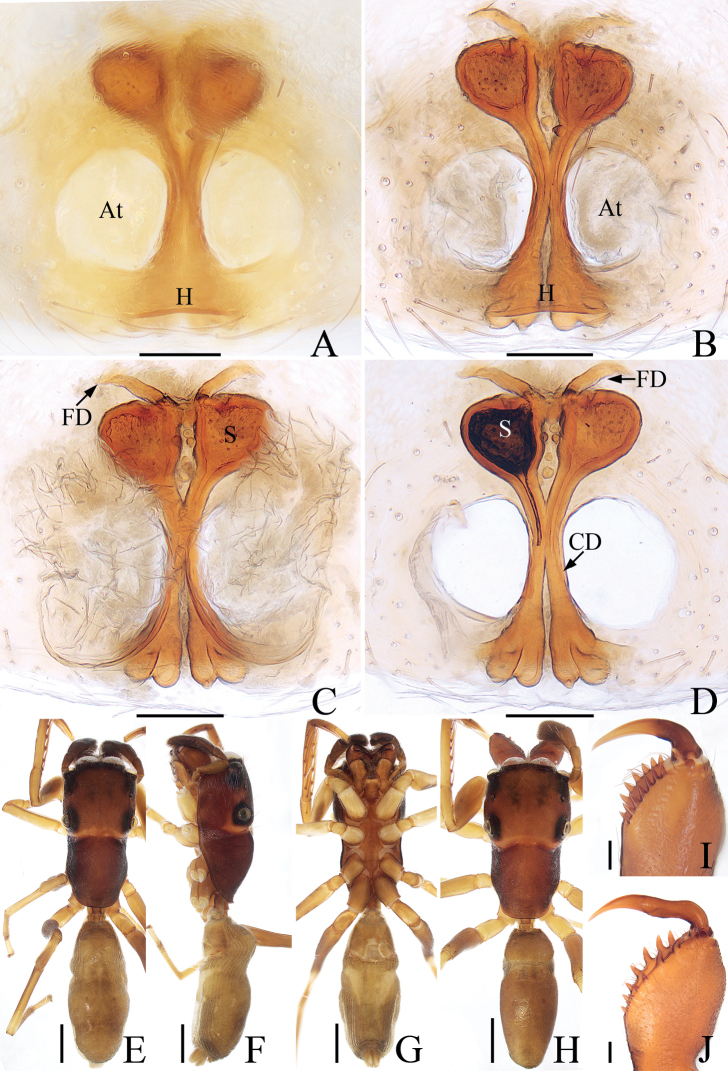
*Toxeushainan* sp. nov., female holotype and male paratype **A, B** epigyne, ventral **C, D** vulva, dorsal **E** holotype habitus, dorsal **F** ditto, lateral **G** ditto, ventral **H** male paratype habitus, dorsal **I** holotype chelicera, posterior **J** male paratype chelicera, posterior. Scale bars: 0.1 mm (**A–D**); 0.2 mm (**I, J**); 1.0 mm (**E–H**). Abbreviations: At – atrium; CD – copulatory duct; FD – fertilization duct; H – epigynal hood; S – spermatheca.

*Toxeushainan* sp. nov. resembles *T.latithoracicus* (Yamasaki & Huang, 2012) known from Ryukyu Island by having short chelicerae in males, tapered embolus, and similarly shaped RTA, but it can be easily distinguished by the following characters: (1) the presence of proximal processes of the sclerotized portions of copulatory ducts (Fig. 18C, D), whereas they are absent in *T.latithoracicus* (Yamasaki and Huang 2012: figs 12, 13); (2) the spermathecae are ca. as long as wide (Fig. 18A–D), whereas they are longer than wide in *T.latithoracicus* (Yamasaki and Huang 2012: figs 12, 13); (3) the RTA is curved inward distally in ventral view (Fig. 17A), whereas it is straight in *T.latithoracicus* (Yamasaki and Huang 2012: fig. 5); (4) the sternum is > 3 × longer than wide (females only) (Fig. 18G), whereas it is < 2.8 × longer than wide in *T.latithoracicus* (Yamasaki and Huang 2012: fig. 11).

##### Description.

**Male** (Figs 17, 18H, J). Total length 6.83 Carapace 3.38 long, 1.97 wide. Abdomen 3.10 long, 1.31 wide. Eye sizes and inter-distances: AME 0.66, ALE 0.35, PLE 0.35, AERW 1.86, PERW 1.98, EFL 1.45. Leg measurements: I 6.89 (2.15, 1.08, 2.05, 1.01, 0.60), II 5.54 (1.65, 0.88, 1.40, 1.01, 0.60), III 5.60 (1.65, 0.75, 1.30, 1.30, 0.60), IV 7.90 (2.35, 0.90, 2.00, 2.00, 0.65). Habitus (Fig. 18H) similar to that of female except with longer chelicerae, covered entirely by dorsal scutum of abdomen. Palp (Fig. 17A–C): tibia > 2 × longer than wide, with tapered RTA curved into S-shape in ventral view, curved towards cymbium distally in retrolateral view, and lamellar flange near the base of RTA; cymbium longer than wide, setose; bulb almost round, with sperm duct extending along prolateral sub-margin; embolus flat, coiled ~ 1.5 ×, tapered distally, and pointed apically.

**Female** (Fig. 18A–D, E–G, I). Total length 7.82 Carapace 3.73 long, 1.91 wide. Abdomen 3.78 long, 1.64 wide. Eye sizes and inter-distances: AME 0.71, ALE 0.41, PLE 0.39, AERW 2.00, PERW 2.05, EFL 1.56. Leg measurements: I 7.01 (2.15, 1.08, 2.13, 1.05, 0.60), II 5.55 (1.65, 0.85, 1.45, 1.00, 0.60), III 6.03 (1.75, 0.80, 1.38, 1.50, 0.60), IV 8.40 (2.35, 1.00, 2.15, 2.15, 0.75). Carapace red-brown, pale yellow in cervical groove, with pair of indistinct dark patches medially on square cephalic region, covered with thin setae. Chelicerae red-brown, with seven teeth on both margins, respectively. Endites > 2 × longer than wide, with dense setae on inner margins of distal 1/2. Labium longer than wide, with dense antero-marginal setae. Sternum fusiform, > 3 × longer than wide. Legs pale to brown, with six and two pairs of ventral macrosetae on tibiae and metatarsi I, respectively. Abdomen elongated, constricted at anterior 2/5, dorsum brown, with indistinct patch medially, covered with thin setae, venter same color as dorsum, with pair of pale patches mediolaterally. Epigyne (Fig. 18A–D): longer than wide, with pair of round atria posteromedially, and inverted, cup-shaped hood posteriorly; sclerotized parts of copulatory ducts originate posteriorly, slightly curved medially, connect with posterior edges of oval spermathecae, with two pairs of proximal processes; fertilization ducts originate from antero-inner edges of spermathecae.

##### Distribution.

Only known from the type locality, Hainan Island, China.

## Supplementary Material

XML Treatment for Irura

XML Treatment for Irura liae

XML Treatment for Irura mii

XML Treatment for Irura pengi

XML Treatment for Marengo

XML Treatment for Marengo ganae

XML Treatment for Marengo zhengi

XML Treatment for Nungia

XML Treatment for Nungia tangi

XML Treatment for Pengmarengo

XML Treatment for Pengmarengo yangi

XML Treatment for Philates

XML Treatment for Philates zhoui

XML Treatment for Toxeus

XML Treatment for Toxeus hainan
